# Changing Trends in Antimicrobial Susceptibility Patterns of Bloodstream Infection (BSI) in Secondary Care Hospitals of India

**DOI:** 10.7759/cureus.37800

**Published:** 2023-04-18

**Authors:** T Karuna, Ayush Gupta, Apurva Vyas, Shweta Kumar, Ananyan Sampath, Pramod Goel, Pankaj Shukla, Vivek Mishra, Sandeep Sharma, Sourabh Chakraborty, Shree Prakash Jaiswal, Abhi Mishra, Apoorwa Gupta, Manisa Sahu, Shreshtha Tiwari, Anisa Pal, Manish Nagendra, Harish Gautham, Kamlesh Patel, Shruti Asati, Sagar Khadanga

**Affiliations:** 1 Microbiology, All India Institute of Medical Sciences, Bhopal, Bhopal, IND; 2 General Medicine, All India Institute of Medical Sciences, Bhopal, Bhopal, IND; 3 Medical School, All India Institute of Medical Sciences, Bhopal, Bhopal, IND; 4 Health Policy, Government of Madhya Pradesh, Bhopal, IND; 5 Microbiology, Bansal Hospital, Bhopal, IND; 6 Microbiology, Choitram Hospital, Indore, IND; 7 Microbiology, Government Prakashchandra Sethi Hospital, Indore, IND; 8 Microbiology, Rajshree Apollo Hospital, Indore, IND; 9 Microbiology, Balco Medical Centre, Raipur, IND; 10 Microbiology, Jabalpur Hospital and Research Centre, Jabalpur, IND; 11 Microbiology, Netaji Subhash Chandra Bose Medical College, Jabalpur, IND; 12 Microbiology, Jai Prakash Hospital, Bhopal, IND; 13 Microbiology, Medanta Hospital, Indore, IND

**Keywords:** bloodstream infections, sepsis, “gram positive bacteria, “enterobacteriaceae”, “antimicrobial stewardship”, microbial”, “drug resistance, “bacteremia”

## Abstract

Introduction

Bloodstream infection (BSI) and subsequent sepsis are life-threatening medical conditions. The onset of antimicrobial resistance and subsequent multi-drug resistant organisms (MDRO) significantly increase healthcare-associated expenditure with adverse clinical outcomes. The present study was undertaken to identify the trends of BSI in community settings in secondary care hospitals (smaller private hospitals and district hospitals) in the state of Madhya Pradesh in Central India with the support of the Indian Council of Medical Research (ICMR) and National Health Mission, Madhya Pradesh.

Methodology

The present study was a prospective, longitudinal observational chart review type of study. The study was carried out at 10 secondary care hospitals (eight smaller private hospitals and two government district hospitals) nominated by the State Government as part of the ICMR Antimicrobial Resistance Surveillance and Research Network (AMRSN). The hospitals were nominated depending on the availability of a microbiology laboratory and a full-time microbiologist.

Result

A total of 6202 blood samples were received from patients with suspected BSI, out of which 693 samples were positive for aerobic culture. Among these, 621 (89.6%) showed bacterial growth and 72 (10.3%) grew Candida species (spp). Out of the 621 bacterial growth samples, Gram-negative bacteria were 406 (65.3%) and Gram-positive bacteria were 215 (34.6%). Among the Gram-negative isolates (406), the predominant isolate was *Escherichia coli *(115; 28.3%) followed by *Klebsiella pneumoniae* (109; 26.8%), *Pseudomonas aeruginosa* (61; 15%), *Salmonella *spp. (52; 12.8%), *Acinetobacter *spp. (47; 11.6%) and the other *Enterobacter *spp. (22; 5.4%). Among the Gram-positive isolates (215), the predominant isolate was *Staphylococcus aureus* (178; 82.8%) followed by *Enterococcus *spp. (37; 17.2%). Among the *Escherichia coli*, third-generation cephalosporin resistance was identified in 77.6%, piperacillin-tazobactam resistance in 45.2%, carbapenem resistance in 23.5% and colistin resistance in 16.5% of cases. Among the *Klebsiella pneumoniae*, third-generation cephalosporin resistance was identified in 80.7%, piperacillin-tazobactam resistance in 72.8%, carbapenem resistance in 63.3% and colistin resistance in 14% of cases. Among the *Pseudomonas aeruginosa*, ceftazidime resistance was identified in 61.2%, piperacillin-tazobactam resistance in 55%, carbapenem resistance in 32.8%, and colistin resistance in 38.3% of cases. Among the *Acinetobacter *spp., piperacillin-tazobactam resistance was identified in 72.7%, carbapenem resistance in 72.3%, and colistin resistance in 9.3% cases. While analyzing the antibiogram for Staphylococcus aureus isolates, methicillin resistance (MRSA) was seen in 70.3% of cases, followed by vancomycin resistance (VRSA) in 8% of cases and linezolid resistance in 8.1%. Among the *Enterococcus* spp. isolates, linezolid resistance was found in 13.5%, vancomycin resistance (VRE) in 21.6%, and teicoplanin resistance in 29.7% of cases.

Conclusion

In conclusion, the first-ever study to identify the risk of high-end antibiotics causing significant drug resistance in secondary and tertiary care settings has highlighted the urgent need for more randomized control studies and proactive measures from healthcare authorities and serves as a beacon for future research efforts and underscores the importance of implementing antibiograms to combat the growing threat of antibiotic resistance.

## Introduction

High-end antimicrobials (and antibiotics) in human health are used for the treatment of sepsis. Inappropriate use of antibiotics is one of the important reasons for the development of anti-microbial resistance (AMR) imparting a significant threat to human health. The global action plan on AMR (GAP-AMR) was designed by World Health Organization (WHO) in 2015 followed by the Indian National Action Plan on AMR (NAP-AMR) 2017 [1-3].

Among the many causes of sepsis, BSI is a serious and potentially life-threatening condition caused by the invasion of microorganisms into the bloodstream. BSI has been more and more recognised across the globe including India over the past couple of years [4,5]. Combined with the ever-increasing antimicrobial resistance (AMR) amongst the MDROs, BSI has become one of the major causes of sepsis and subsequent mortality worldwide [6]. BSI may be hospital-acquired (HA-BSI) which occurs 48 hours after hospitalisation for an unrelated disease, or community-acquired (CA-BSI), without prior history of hospitalisation or within 48 hours of present hospitalisation [7,8].

Bacteria are the most commonly isolated organisms among patients with BSI. Most commonly, Gram-positive bacteria such as *Staphylococcus aureus* and *Streptococcus* species are isolated from BSI followed by Gram-negative bacteria such as *Escherichia coli* and *Klebsiella* species [9,10]. Recently, fungi like *Candida* are also increasingly isolated from patients with BSI [9,11]. Multi-drug resistant strains of these commonly encountered pathogens are isolated with increasing frequency over the last decade, resulting in more treatment failures and adverse clinical outcomes [5,6,12,13].

Most of these studies related to BSI have been from tertiary care centres. There is a paucity of literature about BSI in primary and secondary-level healthcare delivery systems in community settings. It is imperative to identify the trends of BSI in community settings, particularly in developing nations like India. These trends of CA-BSI will be of utmost use in the subsequent development of local antibiogram and antibiotic policies.

Indian Council of Medical Research (ICMR) has initiated a nationwide “Antimicrobial Resistance Surveillance and Research Network” (AMRSN) [14,15]. After the successful consolidation of anti-microbial stewardship programmes (AMSP) at various tertiary-care centres, AMRSN has envisaged extending to secondary-level healthcare systems for consolidation of AMSPs across India. Madhya Pradesh State Action Plan for the Containment of Antimicrobial Resistance (MPSAPCAR) was developed in 2019 on the guidelines of NAP-AMR. The present study was conceived to identify the pattern of antibiograms for BSI in secondary care hospitals (district hospitals/nursing homes) in central India with support from ICMR-AMRSN and MPSAPCAR.

## Materials and methods

**Figure 1:**
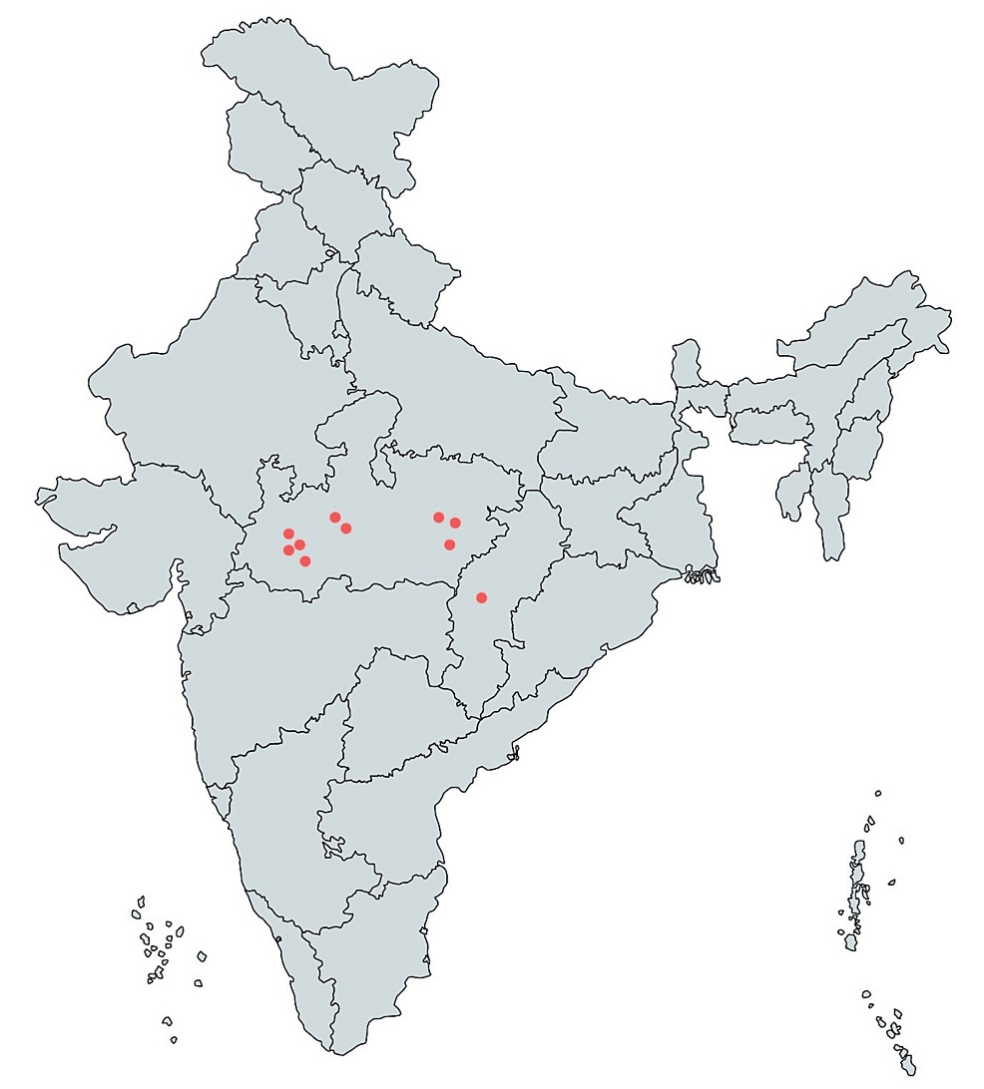
AIIMS Bhopal ICMR-AMRSN initiated network This figure is indicative of all centres selected for our study. Four centres from Indore (Madhya Pradesh), three from Jabalpur (Madhya Pradesh), two from Bhopal (Madhya Pradesh) and one from Raipur (Chhattisgarh)

AIIMS Bhopal is an institute of national importance (INI) in central India and part of ICMR-initiated AMRSN. The ICMR-AMSRN AIIMS Bhopal sub-network runs a regional anti-microbial stewardship program (AMSP) consisting of 10 centres (two government district hospitals and eight nursing homes) in smaller cities of Madhya Pradesh state in India. The location of these cities is presented in Figure 1. The sites were carefully nominated by the Government of Madhya Pradesh and ICMR-AMRSN, on the parameters of the availability of an in-house microbiology laboratory and a full-time microbiologist.

Study setting

Among all the 52 districts of MP, only two district hospitals (DHs) possessed microbiological culture facilities and hence were included in the study. The remaining eight study sites were located in urban/semi-urban areas and were private nursing homes. 

Study design

The current study was a prospective longitudinal observational chart review type of study. Among each secondary care centre, priority areas for AMSP implementation were identified. Clinical data were collected by the nursing officers and the microbiological data were collected by the laboratory technician and verified by the microbiologist. Antimicrobial susceptibility test was carried out using the Kirby-Bauer disc diffusion method as per the Clinical Laboratory Standards Institute (CLSI) guidelines on Muller Hinton agar. The formal sample size was not calculated and planned for consecutive and feasible sampling during the study period. Antibiogram was generated only for those organisms with a cumulative frequency of more than 30 samples. Speciation of *Acinetobacter* spp. and *Enterococcus* spp. isolates could not be done due to the unavailability of resources at these smaller secondary centres. Third-generation cephalosporin resistance for the Enterobacteriaceae family was tested using ceftriaxone and for Pseudomonas using ceftazidime and was calculated by 100 minus the susceptibility percentage of ceftriaxone/ceftazidime. Carbapenem resistance was calculated by 100 minus the susceptibility percentage of meropenem. Methicillin resistance was calculated by 100 minus the susceptibility percentage of oxacillin. Cleaned data were entered in a Microsoft Excel spreadsheet. The data were summarized as frequencies and percentages up to one decimal value. 

Ethical clearance

The study was carried out as part of ICMR-AMRSN, with Institute Human Ethics Committee (IHEC) approval vide Letter No. LOP/2020/EF0157 dated February 24, 2020. As the study was only observational and with data obtained from chart reviews without any patient identifiers, a waiver of consent was granted by IHEC. The present data set was collected from 1st April 2022 to 30 September 2022. The study procedure was in accordance with the principles of the Declaration of Helsinki.

## Results

In the present study, 6202 blood samples were received and processed for culture. Out of 6202 blood samples, 693 (11.2%) samples showed significant pathological growth. Among 693 positive cultures, 621 (89.6%) showed bacterial growth and 72 (10.3%) were *Candida* spp. Out of 621 bacterial growth, Gram-negative bacteria were 406 (65.3%) and gram-positive bacteria were 215 (34.6%)

Among the Gram-negative isolates (406), the predominant isolate was *Escherichia coli*, 115 (28.3%) followed by *Klebsiella pneumoniae*, 109 (26.8%), *Pseudomonas aeruginosa*, 61 (15%), *Salmonella* spp., 52 (12.8%), *Acinetobacter *spp., 47 (11.6%) and the other *Enterobacter *spp., 22 (5.4%). Among the Gram-positive isolates (215), the predominant isolate was *Staphylococcus aureus,* 178 (82.8%) followed by *Enterococcus *spp., 37 (17.2%). 

**Figure 2:**
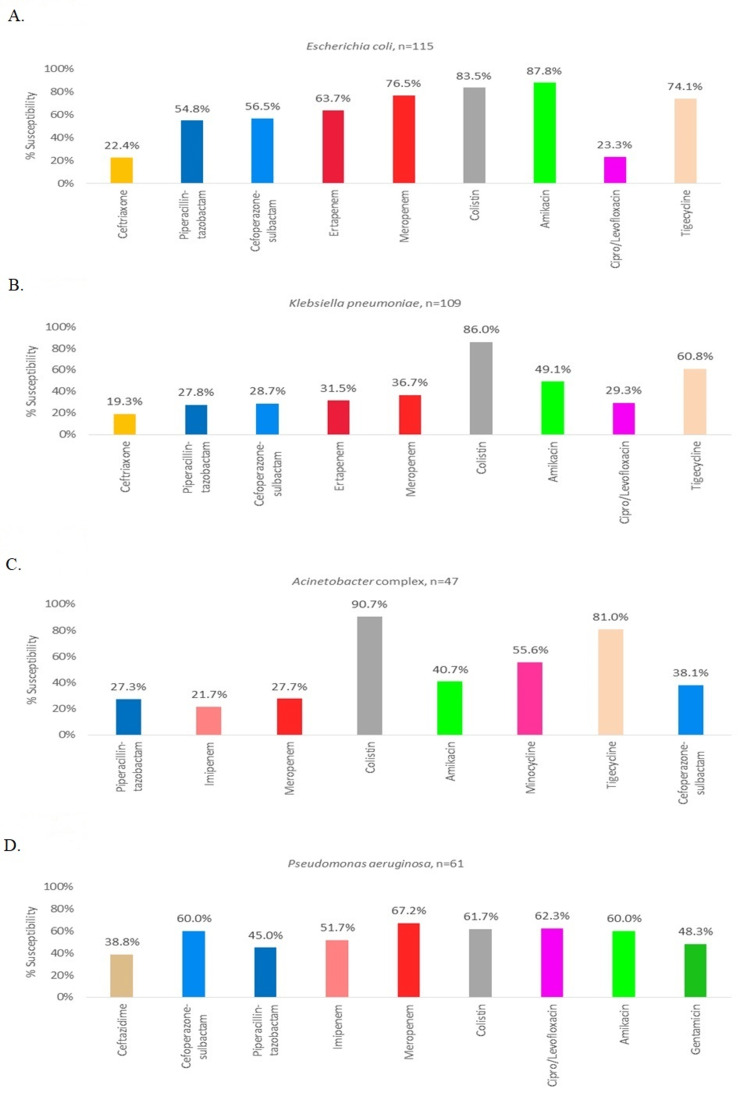
Antibiogram of Gram-negative BSI among secondary care hospitals of central India The antibiogram represents the percentage susceptibility (as depicted on the Y-axis) of the given gram-negative organism with various tested antibiotics (X-axis) in our study. X-axis represents the proportion of total isolated cultures of the given species susceptible to a given agent. (A) depicts the susceptibility pattern of *Escherichia coli* in our study. Most bacteria were resistant to Amikacin, colistin, meropenem, tigecycline with fewer bacteria susceptible to other commonly employed antimicrobial agents as shown. (B) depicts the susceptibility pattern of *Klebsiella pneumoniae*. in our study, where most isolates were susceptible only to colistin and tigecycline. (C) depicts the susceptibility pattern of *Acinetobacter Complex* in our study, where most isolates were susceptible only to colistin and tigecycline. (D) depicts the susceptibility pattern of *Pseudomonas aeruginosa* in our study, where most isolated bacteria were not uniquely susceptible to any specific antimicrobial agent and only around 60% of bacteria were susceptible to any given antimicrobial agent. BSI: bloodstream infection

While analysing the antibiogram for *Escherichia coli*, amikacin was the most susceptible drug with a susceptibility pattern of 87.8%. Resistance to third-generation cephalosporin was noticed in 77.6% of cases, piperacillin-tazobactam resistance in 45.2% of cases, carbapenem resistance in 23.5% and colistin resistance in 16.5% of cases. The detailed antibiogram of *Escherichia coli* is provided in Figure 2.

While analysing the antibiogram for *Klebsiella pneumoniae*, colistin was the most susceptible drug with a susceptibility pattern of 86%. Resistance to third-generation cephalosporin was noticed in 80.7% of cases, piperacillin-tazobactam resistance in 72.8% of cases and carbapenem resistance in 63.3%. The detailed antibiogram of Klebsiella pneumoniae is provided in Figure 2.

While analysing the antibiogram for *Pseudomonas aeruginosa* isolates, meropenem was the most susceptible drug with a susceptibility pattern of 67.2%. Resistance to third-generation cephalosporin (ceftazidime) was noticed in 61.2% of cases, piperacillin-tazobactam resistance in 55% of cases, carbapenem resistance in 32.8% and colistin resistance in 38.3% of cases. The detailed antibiogram of *Pseudomonas aeruginosa* is provided in Figure 2.

While analysing the antibiogram for *Acinetobacter *spp. isolates, colistin was the most susceptible drug with a susceptibility pattern of 90.7%. Resistance to piperacillin-tazobactam was noticed in 72.7% of cases, carbapenem resistance in 72.3% and colistin resistance in 9.3% of cases. The detailed antibiogram of* Acinetobacter* spp. isolates is provided in Figure 2.

**Figure 3:**
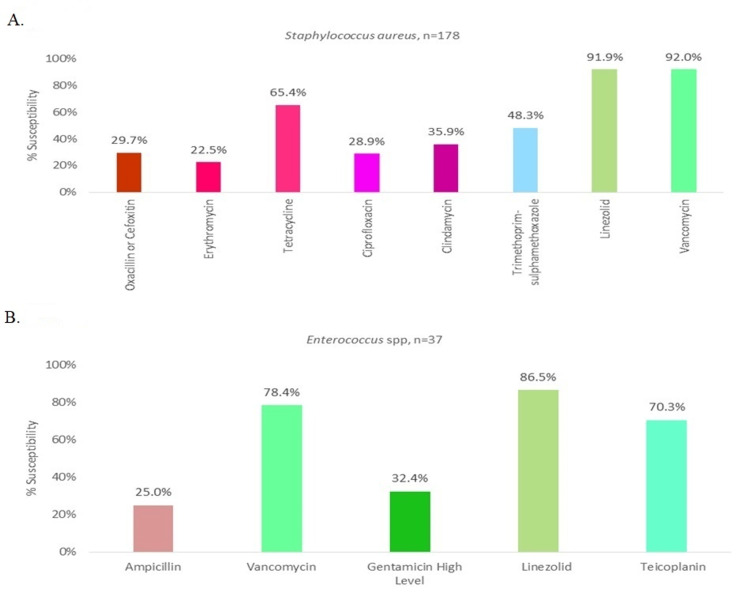
Antibiogram of Gram-positive BSI among secondary care hospitals of central India The antibiogram represents the percentage susceptibility (as depicted on the Y-axis) of the given gram-positive organism with various tested antibiotics (X-axis) in our study. X-axis represents the proportion of total isolated cultures of the given species susceptible to a given agent. (A) depicts the susceptibility pattern of *Staphylococcus aureus *in our study, indicative of the in-vitro efficacy of linezolid and vancomycin. (B) depicts the susceptibility pattern of *Enterococcus *spp. in our study, wherein most isolates were susceptible to linezolid, vancomycin and teicoplanin. BSI: bloodstream infection

While analysing the antibiogram for *Staphylococcus aureus* isolates, methicillin resistance was seen in 70.3% of cases, followed by vancomycin resistance in 8% of cases and linezolid resistance in 8.1%. The detailed antibiogram of *Staphylococcus aureus* isolates is provided in Figure 3. While analysing the antibiogram for *Enterococcus *spp. isolates, it was found that linezolid was the most susceptible drug (86.5%). Teicoplanin resistance was found in 29.7% and vancomycin resistance was found in 21.6% of cases. The detailed antibiogram of *Enterococcus *spp. isolates is provided in Figure 3.

## Discussion

The presence of viable bacteria or fungi in the bloodstream is known as bloodstream infection (BSI) [16]. Subsequent organ dysfunction in BSI leads to the clinical cascade of sepsis. Recently over the last couple of years, BSI and related adverse events have been in the limelight [17]. BSI is one of the most fearsome events in ICU settings and can lead to prolonged hospital stays and subsequent increases in morbidity/mortality, perpetuating a huge financial load on the healthcare system. Our study is unique in its attempt to document the trends of BSI in community settings in central India.

Out of the 6202 patients’ samples, 693 samples (11.2%) were identified as having BSI. The prevalence rate of microbiological BSI or blood culture positivity rate was 11.2% and in line with previous studies from India, as reported by Yangzom et al. (2020), 1340/13,091 (10.2%), Banik et al. (2020), 270/1895 (4.2%) [18,19]. Studies from other parts of the developing world show certain differences and similarities, with prevalence ranging from 11.2% in Bangladesh, 11.7% in Rwanda, 14.2% in Malawi, 20.3% in Iran, and 28.3% in Pakistan [11,19-23]. These varied ranges of the prevalence of BSI may be explained by differences in infrastructure and standard operating procedures of microbiological laboratories. This variation may also be due to many other factors like geographical locations, patient type, age, gender, timing, volume and the number of blood cultures taken and differences in the blood culture system.

The present study observed Gram-negative BSI to be more common than Gram-positive BSI (65.4% vs. 34.6%). Our study was similar to studies from the United States, Rwanda, Bangladesh, Iran and other Indian studies as well [13,18,20,22-24]. However a higher incidence of Gram-positive BSI has been identified by Banik et al. (2018), Nazir et al (2018) Vidyasagar et al. (2019) from India [19,25,26].

Fungi like *Candida* species were isolated in 10.3% of cases among all the microbiological BSI cases. The prevalence of *Candida *species in our study was higher compared to the study conducted in other parts of the subcontinent. Vidyasagar et al. (2019) documented its prevalence as 1% in Karnataka (India), 3.3% by Banik et al. (2018) from Port Blair (India), and 1% by Ejaz et al. (2023) from Pakistan [19,21,26]. Literature from other parts of the world also reveals similar low incidences of fungal BSI with 0.3-0.5 per thousand cases in the United States by Pfaller et al. (2002) and Falagas et al. (2010) in Europe [27,28]. However, Nazir et al. (2018) from North India (Jammu and Kashmir) had also previously documented the prevalence of fungal BSI as 20.9% [25]. This extremely higher prevalence of fungal BSI could probably be due to the higher usage of broad-spectrum antibiotics and invasive devices in the ICU settings of secondary care hospitals.

In the present study, MRSA was reported to be 70.3% and VRSA as 8% at the community level of secondary care hospitals. The MRSA and VRSA BSI rates were higher than earlier studies from ICMR-AMRSN which were 47.4% MRSA and 0% VRSA at various tertiary care hospitals. Similar studies from India by Yangzom et al (2020) and Nazir et al (2018) were indicative of 44.3% MRSA, 6.2% VRSA and 22.20 MRSA and 0% VRSA respectively [18,25]. While comparing to other studies from developing nations in the region, our MRSA and VRSA rates were much higher than Pakistan, China, Iran, South Korea and Africa [20,23,29-31]. The details of these rates in various countries are mentioned in Table 1. This higher rate of MRSA and VRSA in the present study may be explained partially by poor infection control practices in these smaller secondary care hospitals and cross-contamination due to overcrowding. Deviation from standard operating procedures while conducting the culture of these organisms might also be suspected as none of these centres were teaching hospitals and none of them had stringent quality checks. Even with this limitation, the rates of MRSA and VRSA are quite significant and more stringent practices of community-level AMSP is the need of the hour.

**Table 1:** Antibiotic resistance pattern in BSI-derived cultures Carba R – carbapenem resistance; 3rd GCR – third-generation cephalosporin resistance; coli R – colistin resistance; VRE – vancomycin-resistant *Enterococcus*; MRSA – methicillin-resistant *Staphylococcus aureus*; VRSA – vancomycin-resistant *Staphylococcus aureus* All numbers in superscript are indicative of respective study reference numbers. ^Enterococcus faecalis, ^^Enterococcus faecium # Meropenem Resistance ᶿ Ceftriaxone Resistance, † Ceftazidime Resistance, ¶ Cefotaxime Resistance - Data not available

Organism	Known Resistance Patterns	Present Study		ICMR-AMSN [3]		Other Indian Studies [19,25,26]	Saudi Arabia [10]		Iran [20]		China [30]		South Korea [29]		Sub-Saharan Africa [23]		Pakistan [21]	Brazil [31]
Gram-Positive Organisms																		
Staphylococcus aureus	MRSA	70.30%	47.4%		50.00%			-		61.50%		42%		53.20%		33.30%	57.14%	-
	VRSA	8%	0%		0%			-		1.20%		0%		0%		3.70%	0%	-
Enterococcus spp.	VRE	21.60%	5.8%^, 32.3%^^		15%			-		22.20%		1.6%		0.6%^, 34%^^		-	0%	-
Gram-Negative Organisms																		
Escherichia coli	3^rd^ GCR	77.6%ᶿ	91.4%ᶿ, 92.7%^¶^, 97%^†^		100% ᶿ ^¶ †^			73.4%ᶿ		68.2%ᶿ		60.7%^†^, 27.8%^¶^		32.4%^¶^, 11.8%^†^		72.7%ᶿ, 74%^¶^, 50%^†^	65%ᶿ, 65%^¶^	20%ᶿ
	Carba R	23.5%^#^,	48.1%^#^		70%^#^			0%^ #^		-		1.4%^#^		0.2% ^# ^		-	0%^#^	2%^#+^
	Coli R	16.5%	56.90%		0%			0%		-		-		0.2%		-	-	-
Klebsiella pneumoniae	3^rd^ GCR	80.7%ᶿ	89.5%ᶿ, 89.8%^¶^, 91.3%^†^		95.46% ᶿ ^¶ †^			85.3%ᶿ		66%ᶿ		58.7%ᶿ, 48.4%^†^		26.1%^¶^, 21.2%^†^		94.2%ᶿ, 85.5%^†^, 89.3%^¶^	0%ᶿ, 23.52%^¶^	50%ᶿ
	Carba R	63.3%^#^	72.6%^#^		18.2%* ^#^			63.5%^#^		-		34.1%^#^		0.9%^#^		-	29.41%^#^	10% ^# ^
	Coli R	14%	57.5%		0%			17.6%		-		-		0.6%		-	-	-
Pseudomonas aeruginosa	3^rd^ GCR	61.2%^†^	64.3%ᶿ, 50%^¶^, 47.8%^†^		100%^¶ ^ᶿ, 50%^†^			-		93.3%ᶿ		6.7%^†^		14.10%ᶿ		66.7%^†^	64.7%ᶿ, 70.58%^¶^, 76.47%^†^	8.3%^†^
	Carba R	32.8%^#^	44.2%^#^		20% ^#^			-		-		10.5%^#^		18.1%^#^		-	-35.29%^#^	16.7%^#^
	Coli R	38.3%	58.10%		0%			-		-		-		0%		-	-	-
Acinetobacter spp.	Carba R	72.3%^#^	85.8%^#^		31.04%^#^			100% ^# ^		93.5%		70.6% ^#^		92.1%		0%	70%^#^	66.7% ^#^
	Coli R	9.3%	63.1%		0%			0%		0%		-		0%		-	-	-

In the present study, *Enterococci *spp. were most sensitive to linezolid (86.5%) followed by vancomycin (78.4%), gentamycin (32.4%) and ampicillin (25%). The detailed antibiogram is provided in Figure 2. Vancomycin-resistant enterococci (VRE) prevalence in our study was 21.6%, which was similar to previous reports from North India by Nazir et al. (2018) with a 22.2% rate and from Iran by Nobandegani et al. (2020) with a 15% rate [20,25]. The earlier ICMR AMRSN study from the tertiary care centre documented that 0.6% of *Enterococcus faecalis* and 34% of *Enterococcus faecium* were vancomycin-resistant [3]. A detailed comparison of VRE rates in various parts of the world is given in Table 1.

Similar to our study, Gram-negative bacteria, specifically *Klebsiella pneumoniae, Escherichia coli, Acinetobacter* species and *Pseudomonas aeruginosa*, are the primary causative agents of BSI in many other countries as depicted in Table 1. The antimicrobial susceptibility patterns of *Enterobacteriaceae* indicate that *Klebsiella pneumoniae* is more resistant to commonly used antibiotics than *Escherichia coli.* Our isolates of *Escherichia coli *were 77.6% resistant to third-generation cephalosporin, 23.5% were carbapenem-resistant and 16.5% were colistin-resistant. The detailed antibiogram of *Escherichia coli* is given in Figure 3. Our isolates of *Klebsiella pneumoniae* were 80.7% resistant to third-generation cephalosporin, 63.3% were carbapenem-resistant and 14% were colistin-resistant. The detailed antibiogram of *Klebsiella pneumoniae* is given in Figure 3. The detailed comparison of the resistance pattern of *Escherichia coli *and *Klebsiella pneumoniae* to other studies from India and outside is provided in Table 1. More than 75% resistance to third-generation cephalosporin in *Escherichia coli* and *Klebsiella pneumoniae* in secondary care hospitals is alarming and there is a pressing need to devise a robust AMSP in community settings. It is also to emphasize the timely availability of culture sensitivity tests for patients of BSI.

In our study, isolates of *Pseudomonas aeruginosa* were 61.2% resistant to third-generation cephalosporin, 32.8% were carbapenem-resistant and 38.3% were colistin-resistant. The detailed antibiogram of *Pseudomonas aeruginosa* is given in Figure 3. In our study isolates of *Acinetobacter* spp. were 72.3% carbapenem-resistant and 9.3% were colistin-resistant, as presented in Figure 3. The detailed comparison of the resistance pattern of *Pseudomonas aeruginosa* and *Acinetobacter* spp. to other studies from India and outside is provided in Table 1. Carbapenem resistance of >30% in *Pseudomonas aeruginosa* and > 70% in *Acinetobacter spp*. in community settings of secondary care hospitals are frightening and immediate attention to this situation is warranted to the highest level of public health experts.

Limitations

This study had several limitations. The microbiological data collection was from smaller nursing homes and district hospitals without stringent quality adherence. BSIs could not be sub-classified into community-acquired and hospital-acquired. Clinical information on the source of sepsis and the patient’s demographical information was not available and hence stratified data analysis could not be performed.

## Conclusions

The extremely wide range of resistance among the common bacteria in community settings of central India reflects the extremely poor anti-microbial stewardship program (AMSP) in most secondary care hospitals of India. These patterns may be because of the unrestricted use of higher antibiotics for improper situations with inaccurate dosages. It is time to develop quality microbiological laboratories at secondary care hospitals for early detection of bloodstream infection and management to save many precious human lives. In conclusion, the first-ever study to identify the risk of broad-spectrum antibiotics causing significant drug resistance in secondary and tertiary care settings has highlighted the urgent need for more randomized control studies and proactive measures from healthcare authorities. This study serves as a beacon for future research efforts and underscores the importance of implementing antibiograms to combat the growing threat of antibiotic resistance.
